# Impact of complex boundary on the hydrodynamic properties of methane nanofluidic flow via non-equilibrium multiscale molecular dynamics simulation

**DOI:** 10.1038/s41598-022-15323-2

**Published:** 2022-06-30

**Authors:** Chuntao Jiang, Wuming Li, Qingsheng Liu

**Affiliations:** 1 School of Mathematics and Statistics, Xinyang Normal University, Xinyang, 464000 China; 2 School of Mathematics and Information Science, Henan Polytechnic University, Jiaozuo, 454000 China

**Keywords:** Mathematics and computing, Nanoscience and technology, Physics

## Abstract

Understanding the impact of complex boundary on the hydrodynamic properties of methane nanofluidic is significant for production optimization and design of energy-saving emission reduction devices. In the molecule scale, however, the microscopic mechanisms of the influence of the complex boundary on the hydrodynamic characteristics are still not well understood. In this study, a mixture boundary Poiseuille flow model is proposed to study the hydrodynamic properties and explore the molecular mechanisms of confined methane nanofluidic using the Non-equilibrium multiscale molecular dynamics simulation (NEMSMD). In order to investigate the influences of nonslip and rough boundary on hydrodynamic behavior of nanofluidic by the present model in one simulation, the coordinate transformation methods regarding the local symmetry is showed. Simulation results show that the atom number density, velocity and temperature profiles present significant differences near the nonslip boundary and rough wall surface. Moreover, the slip length of methane nanofluidic near the rough boundary decreases with the increasing of the temperature. Furthermore, the viscosity values are calculated by parabolic fit of the local velocity data based on the present model, which demonstrates that the impact of the nonslip boundary on the shear viscosity compared with the experiment result is less than one obtained using the rough boundary. In addition, the local contours of rotational and translational energy are plotted, which show that the rotational and translational energies of nonslip boundary are obvious higher than those of rough boundary. These numerical results are very significant in understanding the impact of complex boundary conditions on hydrodynamic properties in nanofluidic theory and the design of nano-devices.

## Introduction

Methane has emerged as a key source of energy supplement and a vital greenhouse gas^1^. Understanding the hydrodynamic properties of methane is of significant interests from both theoretical research and nanofluidic device perspectives. Over the last several decades, numerical modeling of fluid flow inside micro and nanochannels with different boundary conditions plays a significant role in the optimal design of micro and nanofluidic devices, such as shale gas storage^2^, water purification^3^, drug delivery^4^, and nanomanufacturing^5^. The natures of hydrodynamic properties involved into these devices are predominated by the complex wall–fluid interaction force because of the microstructure of wall surfaces at the micro and nanoscales. Moreover, the continuum fluid theory is incompletely satisfied in nanoscale confined problems, where quantities such as velocity profiles do not remain classical Navier–Stokes (N–S) hydrodynamics^6–8^. Both numerical simulation and experimental studies indicated that the classical hydrodynamics theory was not valid when downsizing to the four times diameters of argon atom^7^ or methane molecule^9–11^. Meanwhile, taking into consideration realistic interest of methane due to the fact that it is an important source of energy and a vital greenhouse gas^12^. Besides, to the best of our knowledge, investigation of the impact of nonslip and rough boundaries on the hydrodynamic properties of nanofluidic is rare. In this study, therefore, we propose the mixture boundary Poiseuille flow model to investigate the influence of boundary conditions on the hydrodynamic properties and explore the molecular mechanism of the confined methane nanofluidic.

Previous investigations of the hydrodynamic properties for nanofluidic were reported by Non-equilibrium molecular dynamic (NEMD) or equilibrium molecular dynamic (MD) simulations for various confined nanofluidics with different atom walls. The momentum transport characteristics of fluid molecules were reviewed by Cao et al.^6^ for micro and nanofluidics at various nanochannel surfaces in micro-/nano-electro-mechanical systems. Some computational simulation techniques were discussed by Xie et al.^13^ to study new thermal physical transport phenomena in the length range from nanoscale to micron. The origin of slip or nonslip boundary conditions was reported by MD simulations focusing on the electrostatic systems, pointing that the electrostatic interaction forces play a significant role for slip or nonslip boundary of the clay nanometer surfaces^14^. Meantime, in order to capture the microscopic details of the heat conduction accurately, Li et al.^15^ developed a hybrid Monte Carlo computational framework to investigate large scale heat conduction mechanisms for ballistic–diffusive systems. Hydrodynamic and structural properties of the argon nanofluidic were systematically studied by Sofos et al.^16–18^ using NEMD simulations. They showed that the transport behavior^16,17^ and velocity profiles^18^ presented asymmetric to the channel centerline within a grooved and a ribbed wall channel. Aminfar et al.^19,20^ employed MD simulation to investigate the atomic behavior and nanoparticles aggregation of liquid–solid nanofluidic flows inside nanochannels, and pointed out that the microstructure and aggregation of nanoparticles were significantly influenced by the interaction force of particles at a specified body driving force^20^. Toghraie et al.^21^ carried out MD simulation to investigate the agglutination characteristic of nanoparticles in a nanochannel. They showed that the agglutination time of Copper nanoparticles is faster than that of Platinum nanoparticles in nanochannel. Subsequently, the group of Toghraie^22–25^ studied the effects of geometrical parameters, various nanochannel wall temperatures and the number of nanoparticles on the local physical property evolution and diffusion characteristic of the confined nanifluidic by using MD simulations. They found that the rough boundary conditions significantly influenced on the molecular mechanism and flow characteristic of Poiseuille flow in a nanochannels. He et al.^26^ used the MD simulation to investigate the mass flux of methane nanofluidic in a rough nanoporous under the pressure gradient. Zhang^27–29^ presented the flow factor approach model to investigate the hydrodynamics properties for nanometer fluid confined in different wall widths and various wall–fluid interactions, and showed that the interaction forces of wall–fluid influenced the hydrodynamics characteristics of confined nanofluidic significantly^28^. The dissipative particle dynamics (DPD) framework was applied to explore the impact of wall cavitations shape on flow feature of micron scale confined fluid by Kasiteropoulou et al.^30^. The study indicated that the density, temperature and pressure approached almost a constant in the center domain of nanochannel and their trait nearby the nanochannel surfaces relied on the cavitations size (or roughness). The influences of mass transport were studied by Yan et al.^31^ using a Lattice Boltzmann method for volatile organic confined inside rough micropore, and indicated that the structure of micropore surfaces had significantly impact on the characteristics of mass transfer. Yu et al.^32^ developed a multiscale Lattice Boltzmann method to explore the transport features for shale gas confined inside rough nanopores. However, the nature characteristics of wall materials were ignored in these researches. To resolve the complex wall–fluid interaction problem, NEMSMD framework was developed by Jiang et al.^33^ to probe into the transport properties, the structural characteristics and flow behaviors for the methane fluid inside the various solid atom wall surfaces. They found that the nanochannel surfaces and interaction force between wall atoms and fluid molecules played a very important role on the nanofluidic flow^9,11,33^, and indicated that the parabolic characteristics between diffusion coefficient values and the inverse of nanochannel width were presented for methane fluid confined inside rough nanometer wall surfaces^9^.

Besides, many research works were implemented for investigating the slip length of methane (shale or natural gas) particularly confined in different nanopores, such as physical experiments^34^ and numerical simulations^1,32,33,35–38^. The slip velocities of methane flow confined in nanochannel were investigated by MD simulations, and showed that the influence of rough nanometer pore surfaces on the slip velocity was rather remarkable in an extremely small Kerogen nanopore^36,39^. The permeability and diffusivity of shale (methane and water) were explored by Chen et al.^40^ using the lattice Boltzmann method for the reconstructed shale, and indicated that the Knudsen diffusion influenced significantly the transport mechanisms of shale gas through the porous geometrical configurations. The transport characteristics of methane inside nanofluidic were studied by Nan et al.^35^ using the NEMD simulation, showed that the nanochannel size played a vital role in the slip length of confined nanofluidic when the pressure decreased to 10 MPa. Mirzaeifard et al^38^ used multiscale modeling to study the influence of interface on the hydrodynamics characteristics for water and methane mixture systems, demonstrating that the interfacial tension decreased slightly with pressure drop or temperature increases when the fluid molecules approached the interface. Understanding the hydrodynamics characteristics has significant interests from both numerical simulation technique and fundamental perspectives for shale gas (Shale gas was a borne remarkable resource^1^). With the develop of numerical simulation technique, methane adsorption behavior confined by organic nanochannel was investigated by Cao et al.^41^ using Monte Carlo and MD technique. It has been presented that the surface adsorption plays a key contribution on the hydrodynamics features of nanofluidic flow inside nanopore^37^. Besides, the slip and nonslip boundary has been generally employed to explore flow characteristic of conventional methane nanometer confined fluid reservoirs^36^. The methane clathrate hydrate and adsorption in nanoporous materials are two independent methods for the methane storage technique in high density and pressure^42^. Majumder et al.^43^ proposed that the slippage of nanopore wall surfaces had an outstanding influence on the flow behavior in carbon nanotubes. For nature methane, it would be of remarkable theoretical interest and realistic value to investigate hydrodynamic characteristics through the nature physical and asymmetric nanochannel. For example, its upper surface comprises nonslip boundary, and the lower surface adopts the nature physical boundary.

Investigating the effects of nonslip and rough boundary on the hydrodynamic behavior of confined methane nanofluidic, one of the new contributions of this study, which may be said to have not been reported in any of the previous work. This is one of the most important issues that the previous researchers have simply eliminated. Moreover, direct experiment investigation of the effect of the mixture boundary (the upper plate is nature physical boundary and the lower boundary conducted by nonslip) on flow behaviors is hard for confined methane nanofluidic. However, it indeed has a very important practical significance for understanding the effective mechanism of boundary on the hydrodynamics for confined methane Poiseuille flow profoundly. In this study, a mixture boundary Poiseuille flow model which has both fruitful results and favorable accuracy is proposed. This model couples the advantage of periodic Poiseulle flow with the nature characteristics of nanochannel flow, including the nonslip boundary domain and the natural physical boundary zone. The influence mechanisms of different boundary conditions on the methane nanofluidic are investigated. Moreover, the impact of the temperature on slip length near the rough boundary is discussed by the proposed model. The viscosity and local viscosity of methane nanofluidic are calculated by using the local velocity fitting technique based on the N–S equation. More importantly, compared with the rough nanochannel flow or the periodic Poiseulle flow, the new modeling can precisely captures the microscopic and hydrodynamic characteristics of the nonslip boundary and that of the rough physical boundary in one NEMSMD simulation.

## Modeling and simulation detail

### Mixture boundary Poiseuille flow modeling

**Figure 1:**
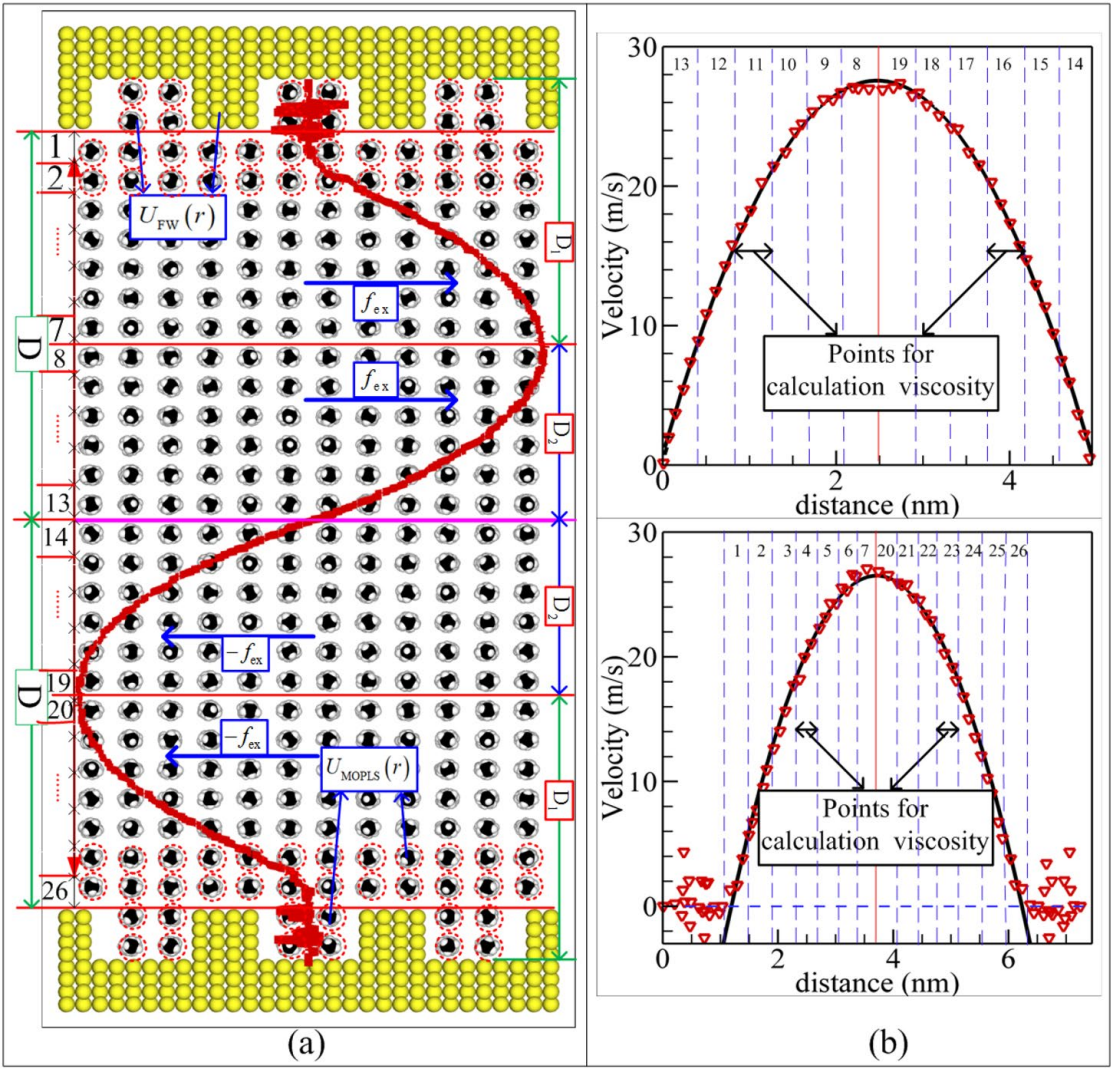
Schematic representations of the mixture boundary Poiseuille flow model (**a**) and the fitted local velocity profile method (**b**). Comparison of the velocity profile of nonslip boundary (top) and that of rough surface (bottom) obtain from one time NEMSMD simulation at given state points (**b**) ($\rho = 377.15\;{\text{kg/m}}^{3} ,\;{\text{T}} = 140\;{\text{K}}$).

In this study, the mixture boundary Poiseuille flow modeling is conducted for dense confined methane nanofluidic using NEMSMD simulation (Fig. 1a). In order to investigate the impact of the nonslip boundary and rough boundary on hydrodynamic properties of confined methane nanofluidic in one simulation, the proposed model coupled the advantage of periodic Poiseulle flow model^44^ and the nature characteristic of nanochannel flow^33^. The original configuration of methane molecule is structured by face centered cubic (FCC) lattice inside two nature silicon atomic wall plates with given roughness. In the directions of x and y coordinate axis, the periodic boundaries are applied for all cases, and 2D is the distance between two rough silicon atomic plates in the *z*–direction. Model dimensions are simulated with dimension of $L_{{\text{x}}} \times L_{{\text{y}}} \times L_{{\text{z}}} = 12.4\;\sigma \times 10.3\;\sigma \times 30.93\;\sigma$ ($\sigma = 4.01\;\text{\AA}$) in order of x, y and z direction vector for simulation state point: ${\text{T}} = 140\;{\text{K}}$, $\rho = 377.15\;{\text{kg/m}}^{3}$. The simulation system consists of ${336}0$ methane molecules, which are confined inside two rough silicon atom walls with $2400$ atoms. For the selected state points, the value of $L_{{\text{x}}}$(or $L_{{\text{y}}}$) is properly resized via the state equation. The mixture boundary Poiseuille flow is determined by using a driving force $f_{{{\text{ex}}}}$ in x direction vector, and the orientation of driving force is opposite when the fluid molecule crosses the centerline (the details see Fig. 1a), the stress and velocity profiles for methane fluid with symmetrical boundary conditions are given by^44,45^

1 $$\tau_{{{\text{zx}}}} = \rho f_{{{\text{ex}}}} \left( {z - \frac{1}{2}{\text{D}}} \right),$$

2 $$\frac{d}{dz}\left( {\eta \frac{{dv_{{\text{x}}} }}{dz}} \right) = - \rho f_{{{\text{ex}}}} .$$

In the above equations, $\tau_{{{\text{zx}}}}$, $f_{{{\text{ex}}}}$, $\rho$, $v_{{\text{X}}}$, $\eta$ and D stand for the shear stress, body driving force, flow density, velocity in streaming, dynamic viscosity and the half of distance between the rough silicon atom plates, respectively. Meantime, the ABAB stacking is applied to construct the silicon atom for nanochannel plates due to the fact that it is an equilibrium configuration^46^. To quantify the roughness of natural physical wall surfaces, the rough nanochannel surfaces are constructed by using the periodic rectangular wave^33^

3 $$\Delta z(x) = \left\{ {\begin{array}{*{20}c} {2A,} & {0 < x \le {\lambda \mathord{\left/ {\vphantom {\lambda {2,}}} \right. \kern-\nulldelimiterspace} {2,}}} \\ {0,} & {{\lambda \mathord{\left/ {\vphantom {\lambda 2}} \right. \kern-\nulldelimiterspace} 2} < x \le \lambda ,} \\ \end{array} } \right.$$

where $A$ is the amplitude, $\lambda$ indicates the wavelength.

To explore the effect mechanism of boundary condition on the methane nanofluidic, the nonslip boundary is applied in the periodic Poiseuille flow, and the distance between two rough plates is $2{\text{D = 30}}{.93}\;\sigma \;(\sigma = 4.01\;\text{\AA})$ and the rough configuration ($\lambda = 4.31\;\sigma ,\;A{ = }1.07\;\sigma$) is adopted. In driving force direction (i.e. x-direction), an integer times of wavelength $\lambda$ is employed to match the boundary condition of its periodicity. All interaction forces between methane molecules are conducted by our previous modification of optimized potential for liquid simulation (MOPLS) model in this study, because it could estimate accurately the mass transfer behaviors and structural characteristic for methane nanofluidic by the previous study of Jiang et al.^47^. It is defined as

4 $$U_{{{\text{MOPLS}}}} \left( {r_{ij} } \right) = \sum\limits_{\alpha ,\beta } {\sum\limits_{i \in \alpha ,j \in \beta } {\left( {4\varepsilon_{ab} \left( {\left( {{{\sigma_{ab} } \mathord{\left/ {\vphantom {{\sigma_{ab} } {r_{ij} }}} \right. \kern-\nulldelimiterspace} {r_{ij} }}} \right)^{12} - \left( {{{\sigma_{ab} } \mathord{\left/ {\vphantom {{\sigma_{ab} } {r_{ij} }}} \right. \kern-\nulldelimiterspace} {r_{ij} }}} \right)^{6} } \right) + {{q_{i} q_{j} } \mathord{\left/ {\vphantom {{q_{i} q_{j} } {4\pi \varepsilon_{0} r_{ij} }}} \right. \kern-\nulldelimiterspace} {4\pi \varepsilon_{0} r_{ij} }}} \right)} } ,$$

where $\varepsilon$ and $\sigma$ denote the energy well of methane molecule and length unit, respectively, $q$ is point charge, $\varepsilon_{0}$ indicates the permittivity of vacuum, and $r_{ij}$ denotes the interaction distance between atoms (C or H) of methane, $a$ and $b$ are employed to distinguish the atom of C and H, $\alpha$ and $\beta$ mark different methane molecules. In methane model: $l_{{{\text{CH}}}} = 1.087\;\text{\AA}$ and ${\kern 1pt} q_{{\text{C}}} = - 4q_{{\text{H}}} = - 0.572\;e$($e = 4.803 \times 10^{ - 10} \;{\text{esu}}$). The details of MOPLS model are listed below (see Table 1).

**Table 1:** The details of MOPLS model adopted in this study^47^.

TYPE	Parameter	C–C	C–H	H–H
MOPLS	${\varepsilon \mathord{\left/ {\vphantom {\varepsilon {k_{B} }}} \right. \kern-\nulldelimiterspace} {k_{B} }}\left( {\text{K}} \right)$	$46.8$	$17.17$	$6.30$
	$\sigma \left( \text{\AA} \right)$	$3.45$	$3.06$	$2.67$

The NEMSMD framework is employed to manipulate the interaction force between wall atom and methane molecule (refer to Fig. 1), i.e. it is determined by coupling the CG potential of methane and silicon atom potential based on L–B mixing rule when the methane molecule arrives near the wall ($\le r_{{{\text{cut}}}}$). The mainly advantage not only observe atomic information using the NEMSMD simulation, but also can ensure the interaction between wall atom and fluid molecule approach the realistic interaction of wall–fluid. The validity of NEMSMD framework can be referred to literature^10,33^. Furthermore, the CG potential model should be consistent with the potential function of silicon. In this paper, the LJ (12–6) potential is applied to calculate the interaction between two silicon atoms. Thus, the interaction potential function between methane molecule and wall atom is showed by

5 $$U_{{{\text{MS}}}} \left( {r_{ij} } \right) = 4\varepsilon_{{{\text{MS}}}} \left( {\left( {\frac{{\sigma_{{{\text{MS}}}} }}{{r_{ij} }}} \right)^{12} - \left( {\frac{{\sigma_{{{\text{MS}}}} }}{{r_{ij} }}} \right)^{6} } \right),$$

where the parameter $\varepsilon_{{{\text{MS}}}}$ and $\sigma_{{{\text{MS}}}}$ are calculated by L–B mixing rule

6 $$\varepsilon_{{{\text{MS}}}} = \chi \sqrt {\varepsilon_{{{\text{CG}}}} \cdot \varepsilon_{{\text{S}}} } ,\,\sigma_{{{\text{MS}}}} = {{\left( {\sigma_{{{\text{CG}}}} + \sigma_{{\text{S}}} } \right)} \mathord{\left/ {\vphantom {{\left( {\sigma_{{{\text{CG}}}} + \sigma_{{\text{S}}} } \right)} 2}} \right. \kern-\nulldelimiterspace} 2}.$$

In this equation, the interaction factor $\chi = 1.5$ is employed in all simulation cases. The interaction potential parameters between two silicon atoms are $\varepsilon_{{\text{S}}} = 1.6885\;{\text{kJ/mol}}$ and $\sigma_{{\text{S}}} = 3.826\;\text{\AA}$, the corresponding details can be seen in the literature^46^. The parameters of CG methane potential $\sigma_{{{\text{CG}}}}$ and $\varepsilon_{{{\text{CG}}}}$ are optimized by the relative entropy minimization framework^48^ for all atom MOPLS in the bulk ensemble of methane under the selected state conditions. The details of optimized parameter for methane CG potential can be referred to our previous literature^49^. The parameter details are showed in Table 2.

**Table 2:** The corresponding details of CG methane potential function.

State points		CG parameter		State points		CG parameter	
${\text{T}}\left( {\text{K}} \right)$	$\rho \left( {{\text{kg/m}}^{3} } \right)$	$\sigma_{{{\text{CG}}}} \;(\text{\AA})$	$\varepsilon_{{{\text{CG}}}} \;\left( {{{{\text{kJ}}} \mathord{\left/ {\vphantom {{{\text{kJ}}} {{\text{mol}}}}} \right. \kern-\nulldelimiterspace} {{\text{mol}}}}} \right)$	${\text{T}}\left( {\text{K}} \right)$	$\rho \left( {{\text{kg/m}}^{3} } \right)$	$\sigma_{{{\text{CG}}}} \;(\text{\AA})$	$\varepsilon_{{{\text{CG}}}} \;\left( {{{{\text{kJ}}} \mathord{\left/ {\vphantom {{{\text{kJ}}} {{\text{mol}}}}} \right. \kern-\nulldelimiterspace} {{\text{mol}}}}} \right)$
$100$	$439.02$	$3.605$	$1.377$	$150$	$358.19$	$$\begin{array}{*{20}l} {3.662} \hfill \\ \end{array}$$	$$\begin{array}{*{20}l} {1.226} \hfill \\ \end{array}$$
$110$	$424.97$	$3.611$	$1.434$	$160$	$336.59$	$3.667$	$1.221$
$120$	$410.13$	$$\begin{array}{*{20}l} {3.625} \hfill \\ \end{array}$$	$$\begin{array}{*{20}l} {1.407} \hfill \\ \end{array}$$	$170$	$310.77$	$3.678$	$1.185$
$130$	$394.29$	$3.636$	$1.329$	$180$	$276.58$	$$\begin{array}{*{20}l} {3.691} \hfill \\ \end{array}$$	$1.120$
$140$	$377.15$	$3.645$	$1.329$	$190$	$200.46$	3.694	1.118

### Simulation details

An equilibrium configuration ABAB stacking is employed for rough silicon atom walls, all atoms are conducted by the harmonic potential site ${\mathbf{r}}_{{{\text{eq}}}}$^50^,

7 $$u\left( {\left| {{\mathbf{r}}\left( t \right) - {\mathbf{r}}_{{{\text{eq}}}} } \right|} \right) = \frac{1}{2}k_{{\text{w}}} \left( {\left| {{\mathbf{r}}\left( t \right) - {\mathbf{r}}_{{{\text{eq}}}} } \right|} \right)^{2} ,$$

here ${\mathbf{r}}\left( t \right)$ is the position coordinate of silicon atom at time $t$. The second derivative value of silicon atom potential at $r = r_{0} = 2^{{{1 \mathord{\left/ {\vphantom {1 6}} \right. \kern-\nulldelimiterspace} 6}}} \sigma_{{\text{S}}}$ is used to determine the $k_{{\text{w}}} \left( { = 72\varepsilon_{{\text{S}}} /\left( {2^{{{1 \mathord{\left/ {\vphantom {1 3}} \right. \kern-\nulldelimiterspace} 3}}} \sigma_{{\text{S}}}^{2} } \right)} \right)$. In the direction of x coordinate axis, the body driving force ($f_{{{\text{ex}}}} = 0.075\;\left( {{\varepsilon \mathord{\left/ {\vphantom {\varepsilon \sigma }} \right. \kern-\nulldelimiterspace} \sigma }} \right)$) of methane molecules is employed to refrain the compressibility effects within the linear regime for confined methane nanofluidic^10,51^. The cutoff radius ($r_{{{\text{cut}}}} = 2.5\;\sigma$) is used to calculate the interaction force of different particles for all simulations. Moreover, the fourth Predictor Corrector method is employed to manipulate the motion of mass center for methane fluid molecule. The NVT ensemble is used to command the temperature of simulation system by Nosé–Hoover thermostat^45^. In order to control the simulations system's temperature and not effect on the flow dynamics, the thermostat is coupled to the thermostat in y and z directions, i.e. ${{mv_{{\text{y}}}^{2} } \mathord{\left/ {\vphantom {{mv_{{\text{y}}}^{2} } 2}} \right. \kern-\nulldelimiterspace} 2} = {{k_{B} T} \mathord{\left/ {\vphantom {{k_{B} T} 2}} \right. \kern-\nulldelimiterspace} 2}$ and ${{mv_{{\text{z}}}^{2} } \mathord{\left/ {\vphantom {{mv_{{\text{z}}}^{2} } 2}} \right. \kern-\nulldelimiterspace} 2} = {{k_{B} T} \mathord{\left/ {\vphantom {{k_{B} T} 2}} \right. \kern-\nulldelimiterspace} 2}$, which can be referred to Bhadauria's research^52^ (where $m$, $v_{{\text{y}}}$, $v_{{\text{z}}}$, $k_{B}$ and $T$ are the methane molecule mass, velocity of *y* and *z* directions, Boltzmann constant and simulation temperature, respectively). Although it is not coupled to the velocity of x direction explicitly, this technique can ensure that the thermostat effect is obtained in each direction through intermolecular interactions. In this study, $\sigma = 4.01{\kern 1pt} \;\text{\AA}$ and ${\varepsilon \mathord{\left/ {\vphantom {\varepsilon {k_{B} }}} \right. \kern-\nulldelimiterspace} {k_{B} }} = 142.87\;{\text{K}}$ are the reduced unit, respectively. The total calculation steps are $8.0 \times 10^{5}$. The time step is $\Delta t = 0.001$ ($1.3 \times 10^{ - 15} \;{\text{s}}$). To collect the flow velocity, atom number density and temperature distributions of the confined methane nanofluidic accurately, the original calculation steps $3.0 \times 10^{5}$ are discarded. Furthermore, the motion information of methane fluid molecule is sampled by next $5.0 \times 10^{5}$ calculation steps. The velocity profiles and atom (C and H) number density distribution are collected via dividing the range in z-direction ($2{\text{D}} + 2A$) by $900$ bins. The velocity profiles are collected by^10,16^

8 $$v_{{\text{x}}} \left( z \right) = \left\langle {\frac{{\sum\limits_{i = 1}^{{N_{{{\text{bin}}}} }} {v_{{{\text{x}},i}} \left( {z,\;z + h_{{{\text{bin}}}} } \right)} }}{{N_{{{\text{bin}}}} \left( {z,\;z + h_{{{\text{bin}}}} } \right)}}} \right\rangle ,$$

in order to calculate the velocity profiles of the nonslip boundary ($v_{N,x} \left( {z_{1} } \right)$) and rough boundary ($v_{R,x} \left( {z_{2} } \right)$) by using results of Eq. (8), the coordinate transformation methods on the local symmetry is given by

9 $$v_{{N,{\text{x}}}} \left( {z_{1} } \right) = \left\{ {\begin{array}{*{20}c} {v_{{\text{x}}} \left( {z_{1} + \left( {{\text{D}}_{1} + {\text{D}}_{2} } \right)} \right),} & {0 \le z_{1} \le {\text{D}}_{2} } \\ { - v_{{\text{x}}} \left( {{\text{D}}_{1} - {\text{D}}_{2} + z_{1} } \right),} & {{\text{D}}_{2} < z_{1} \le 2{\text{D}}_{2} ,} \\ \end{array} } \right.$$

10 $$v_{{R,{\text{x}}}} \left( {z_{2} } \right) = \left\{ {\begin{array}{*{20}c} { - v_{{\text{x}}} \left( {z_{2} } \right),} & {0 \le z_{2} \le {\text{D}}_{1} } \\ {v_{{\text{x}}} \left( {z_{2} + \left( {{\text{D}}_{1} + {\text{D}}_{2} } \right)} \right),} & {{\text{D}}_{1} < z_{2} \le 2{\text{D}}_{1} ,} \\ \end{array} } \right.$$

where $$v_{{\text{x}}} \left( {{\text{D}}_{1} } \right) = \min \left\{ {v_{{\text{x}}} \left( z \right)} \right\}$$ or $$v_{{\text{x}}} \left( {2{\text{D}} + 2A - {\text{D}}_{1} } \right) = \max \left\{ {v_{{\text{x}}} \left( z \right)} \right\}$$ for any $$z$$$$\left( {0 \le z \le 2\left( {\text{D + A}} \right)} \right)$$, and $${\text{D}}_{2} = {\text{D}} - {\text{D}}_{1}$$. The number density profiles of C and H atoms are determined by^33^

11 $$N_{{\text{C/H}}} \left( z \right) = \frac{{\left\langle {N_{{\text{bin,C/H}}} \left( {z,\;z + h_{{{\text{bin}}}} } \right)} \right\rangle }}{{\overline{N}}},$$

and the temperature profiles across the fluid regions are calculated by^33^

12 $$T\left( z \right) = \frac{m}{{3Nk_{{\text{B}}} }}\sum\limits_{i = 1}^{N} {\left( {{\mathbf{v}}_{i} \left( z \right) - {\overline{\mathbf{v}}}} \right)^{2} } ,$$

where ${\overline{\mathbf{v}}}$ and ${\mathbf{v}}_{i}$ indicate the velocity of macroscopic flow and velocity of molecule *i*, respectively. $N$ is the total molecule number.

The shear viscosity is determined by the Poiseuille flow method for the methane nanofluidic, Eq. (2) can be reformulated as follows^10^

13 $$\eta = - {{\rho f_{ex} } \mathord{\left/ {\vphantom {{\rho f_{ex} } {\frac{{{\text{d}}^{2} v_{{\text{x}}} \left( z \right)}}{{{\text{d}}z^{2} }}}}} \right. \kern-\nulldelimiterspace} {\frac{{{\text{d}}^{2} v_{{\text{x}}} \left( z \right)}}{{{\text{d}}z^{2} }}}},$$

in addition, the streaming velocity profile $v_{{\text{x}}} \left( z \right)$ is rewritten as

14 $$v_{{\text{x}}} \left( z \right) = v_{0} \left( {z_{0} } \right) + k\left( {z - z_{0} } \right)^{2} ,$$

the local viscosity is calculated by

15 $$\eta_{{l{\text{ay}}}} = - {{\rho f_{ex} } \mathord{\left/ {\vphantom {{\rho f_{ex} } {2k_{{{\text{lay}}}} }}} \right. \kern-\nulldelimiterspace} {2k_{{{\text{lay}}}} }},$$

16 $$k_{{{\text{lay}}}} = {{\left( {v_{{\text{x}}}^{{{\text{lay}}}} - v_{{0}}^{{{\text{lay}}}} } \right)} \mathord{\left/ {\vphantom {{\left( {v_{{\text{x}}}^{{{\text{lay}}}} - v_{{0}}^{{{\text{lay}}}} } \right)} {\left( {z_{{{\text{lay}}}} - z_{0} } \right)}}} \right. \kern-\nulldelimiterspace} {\left( {z_{{{\text{lay}}}} - z_{0} } \right)}}^{2} ,$$

and the shear viscosities for the whole fluid domain of nonslip or rough boundary are determined by

17 $$\eta = \frac{1}{Num}\sum\limits_{lay = 0}^{Num} {\eta_{lay} } ,$$

in the above Eqs. (13–17), $\eta$ denotes the shear viscosity, $v_{0}$ and $z_{0}$ represent the maximum velocity and its position, and $k$ is calculated via the fitted local velocity information derived from the proposed model. It is noted that the fitted local velocity technique will breakdown while the fluid molecules are confined inside the small width (less than 4 times of molecule diameters, the classical N–S equation fails for simple fluids^7,9^) of nanochannels. In this study, we divided 26 layers in the z-direction (Fig. 1a) to calculate the shear viscosity and corresponding local values for methane fluid. Moreover, the collected local velocity samples are shown in Fig. 1b for calculating the local viscosity of the nonslip ($Num = {12}$) and rough ($Num = {14}$) boundary conditions in detail, i.e., the local viscosity of the conducted domain of the nonslip boundary is determined by the fitted local velocity (see from the top of Fig. 1b), and local shear viscosity of the conducted domain of the rough boundary is calculated via the fitted local velocity profiles (refer to the bottom of Fig. 1b). Finally, to study molecular mechanism of confined methane nanofluidic flowing the nonslip and natural physical (silicon atomic plates) boundary conditions, the whole nanochannel is divided into $n_{{\text{x}}} \times n_{{\text{y}}} \times n_{{\text{z}}} = (36 \times 36 \times 90)$ bins with each volume $V_{{{\text{bin}}}} = \left( {{{L_{{\text{x}}} } \mathord{\left/ {\vphantom {{L_{{\text{x}}} } {n_{{\text{x}}} }}} \right. \kern-\nulldelimiterspace} {n_{{\text{x}}} }}} \right) \times \left( {{{L_{{\text{y}}} } \mathord{\left/ {\vphantom {{L_{{\text{y}}} } {n_{{\text{y}}} }}} \right. \kern-\nulldelimiterspace} {n_{{\text{y}}} }}} \right) \times \left( {{{\left( {L_{{\text{z}}} + 4A} \right)} \mathord{\left/ {\vphantom {{\left( {L_{{\text{z}}} + 4A} \right)} {n_{{\text{z}}} }}} \right. \kern-\nulldelimiterspace} {n_{{\text{z}}} }}} \right)$. The velocity field is calculated by^10^

18 $$\left\langle {{\mathbf{v}}_{{{\text{bin}}}} } \right\rangle = \frac{1}{{n_{bin} }}\sum\limits_{i = 1}^{{n_{bin} }} {{\mathbf{v}}_{i} } .$$

The three-dimensional distributions of translational and rotational kinetic energies are respectively calculated by the formula:

19 $$W_{{{\text{Tran,}}\;{\text{bin}}}} = \frac{1}{2}m_{{{\text{bin}}}} \left\langle {{\mathbf{v}}_{{{\text{bin}}}} } \right\rangle^{2} ,$$

and

20 $$W_{{{\text{Rot,}}\;{\text{bin}}}} = \frac{1}{2}\sum\limits_{{\text{x}}} {I_{{\text{x}}} \left\langle {w_{{{\text{x,}}\,{\text{bin}}}} } \right\rangle ^{2} } ,$$

where $m_{{{\text{bin}}}} = mn_{{{\text{bin}}}}$, $I_{{\text{x}}}$, $\left\langle {{\mathbf{v}}_{{{\text{bin}}}} } \right\rangle$ and $\left\langle {w_{{\text{x, bin}}} } \right\rangle$ are mass, one component of the (diagonal) inertia tensor ($\sum\nolimits_{{\text{x}}} {}$ indicate the number for x, y and z coordinate directions), average velocity, and average angular velocity with corresponding coordinate directions. And ${\mathbf{v}}_{i}$ denotes the i-th molecule's velocity, $n_{{{\text{bin}}}}$ is the number of molecule in the bin. All the codes of program are developed by C++ based on Literature^45^, running on windows operating systems.

## Results and discussion

To verify and examine the validity of mixture boundary Poiseuille flow model, the width of nanochannel is selected properly. Meanwhile, the atom number density, velocity and shear stress profiles are calculated. Moreover, the slip length is calculated near the rough boundary. To explore the impact mechanism of the asymmetric boundary condition (with nonslip and rough boundary) on methane nanofluidic, the local shear viscosity, the translational and rotational energies are discussed.

### Density distribution, shear stress, velocity profile and slip length

**Figure 2:**
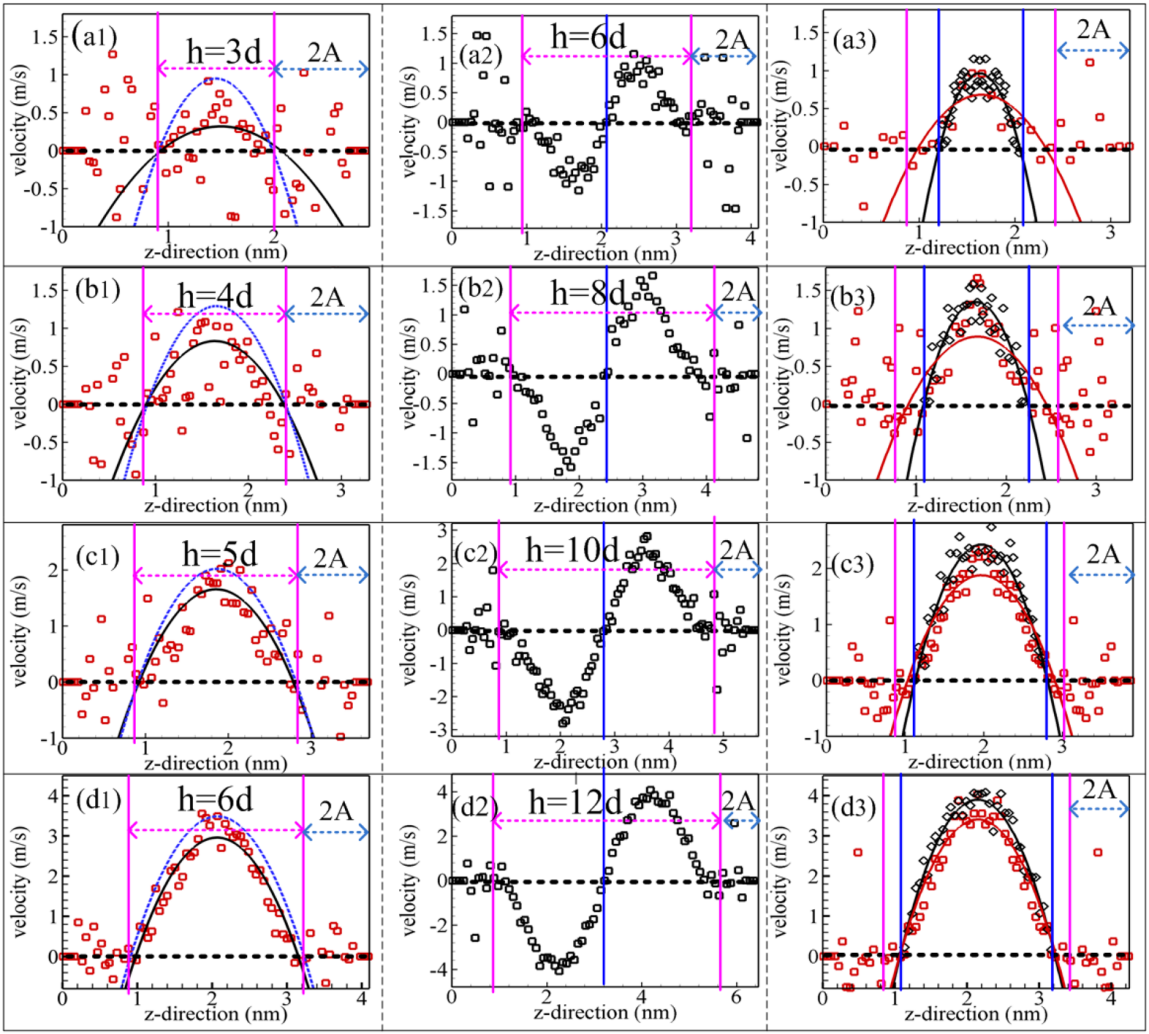
The average velocity profiles (red or black square) are calculated by using the NEMSMD simulations, the red and black solid lines are fitted by the simple points fitting simple points inside the nanochannel, and blue Dash line indicates the analytical results from the Poiseuille flow with the nonslip boundary. Meantime, blue and pink perpendicular lines denote rough and non-slip boundary, respectively.

In order to show the validity and availability of the proposed modeling in studying the hydrodynamic properties for confined methane nanofluidic, the width of nanochannel is selected properly^7,55^. Figure 2 plotted the velocity profiles of methane nanofludic confined inside rough nanochannel wall with various widths (the distance in *z*-direction) and their comparisons with the analytical results from the Poiseuille flow with the non-slip boundary. Figure 2a1–d1 indicate that the fitted velocity profiles (black solid line is fitted by velocity simple points inside the nanochannel based on the NEMSMD simulations) gradually approach the analytical solution (blue Dash line) of the equation of Poiseuille flow (i.e., the analytical solution is obtained from the N–S equations: $v_{x} \left( z \right) = \frac{{\rho f_{{{\text{ex}}}} h}}{2\eta }\left( {\frac{1}{4} - \left( {z - \frac{1}{2}} \right)^{2} } \right)$, referred to literature^45^, where, the viscosity value ($\eta$) obtained from the literature^47^) with the increasing of the width of nanochannel. This simulation results demonstrate that the confined methane nanofluidic gradually presents the feature of Poiseuille flow when the width of nanochannel is bigger than $1.656\;{\text{nm}}$ (about four times methane molecule's diameter). And this results are in good agreement with the argon confined inside nanochennel^7^. Figure 2a3–d3 are obtained from the coordinate transformation method (Eqs. 9–10) based on NEMSMD simulations using the proposed modeling (seen from Fig. 2a2–d2). The results show that the velocity profiles of the fluid domain approaching nonslip boundary are in good agreement with the analytical solution of the Poiseuille flow. And the results of the fluid domain nearby nonslip boundary are in better agreement with the analytical solution of the Poiseuille flow than that of the rough nanochannel (using a single value of external force in an x-direction). Figure 2d3 presents the velocity profiles agree with the analytical solution of the Poiseuille flow, which demonstrate that the distance of between two rough nanochannel walls is larger than 12 molecules diameter (about $4.968\,{\text{nm}}$) if you want to calculate the viscosity by the fitted velocity profile method using the proposed modeling. Therefore, in the study, the distance $2{\text{D = 30}}{.93}\;\sigma$ (about $12\,{\text{nm}}$) between two rough nanochannel walls is selected to study the impact of boundary on the hydrodynamic properties of methane nanofluidic.

**Figure 3:**
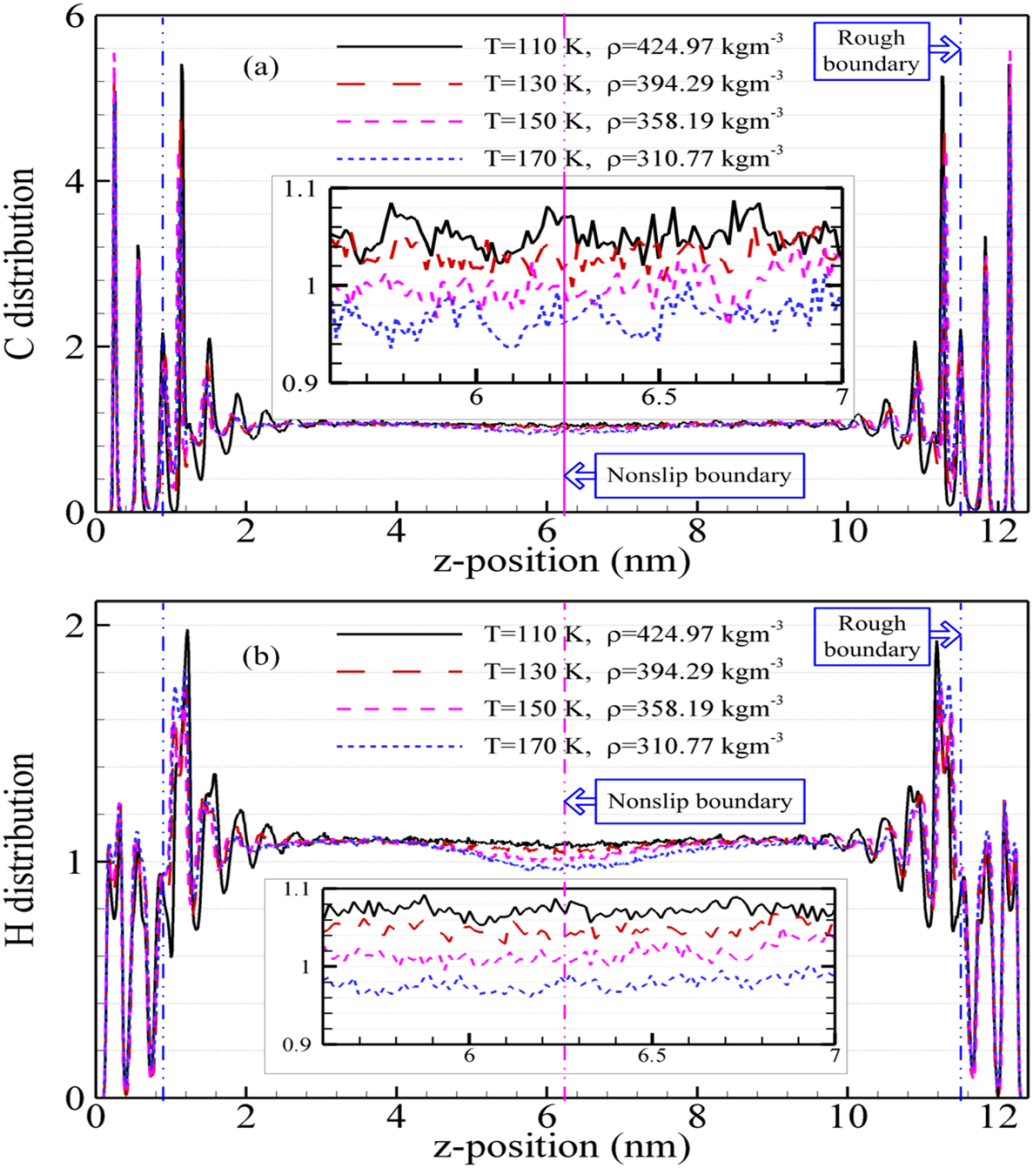
The atoms number density distribution (C (**a**) and H (**b**)) from mixture boundary Poiseuille flow model with different state points.

The C and H atoms number density distribution profiles of methane are showed in Fig. 3 by NEMSMD simulations for different state points. It is obvious that the rough nanochannel surfaces and nonslip boundary have a significant influence on C and H atoms number density distribution when the fluid molecule moves to the boundary domain (nonslip and rough boundary). For different state points, the atoms number density distributions present strong oscillations near the rough nanochannel surfaces, and the methane fluid molecules remain extended period in some layers parallel to the nanochannel surfaces. These layers of the atom number density profiles are situated at the peaks visible because the methane fluid molecule suffered from the stronger repulsion force near the rough nanochannel surfaces ($\le r_{{{\text{cut}}}}$). Moreover, the local atom (C or H) number density reduces with the temperature increase (or the density decrease) while they go into the fluid domain near the nonslip boundary. The values of atom number density approach a certain constant in the center of fluid domain between nonslip and rough boundary. Comparison of the H atom number density with that of C atom is showed. The positions of first peak value for the H and C atom number distribution indicate that the H atom can be firstly observed in the domain of near the nanochannel surfaces. The positions of the second valley and peak for the C atom and that of the third peak and valley for H atom present something similarly. Furthermore, the effect of the nonslip boundary on C atom number density is not more obvious than that of H atom, because the H atom is four times as many as C atom. This result also indicates that the spatial structure of methane is central symmetry spatial tetrahedral. Besides, the number density profile of atom (H or C) near the nonslip boundary is smaller than that of center fluid regions. The reason may be that the methane fluid molecule suffers from different shear forces (or shear-thinning). And the significant degree of this phenomenon depends on the state points, i.e., it increases with density decreasing (or temperature increasing).

**Figure 4:**
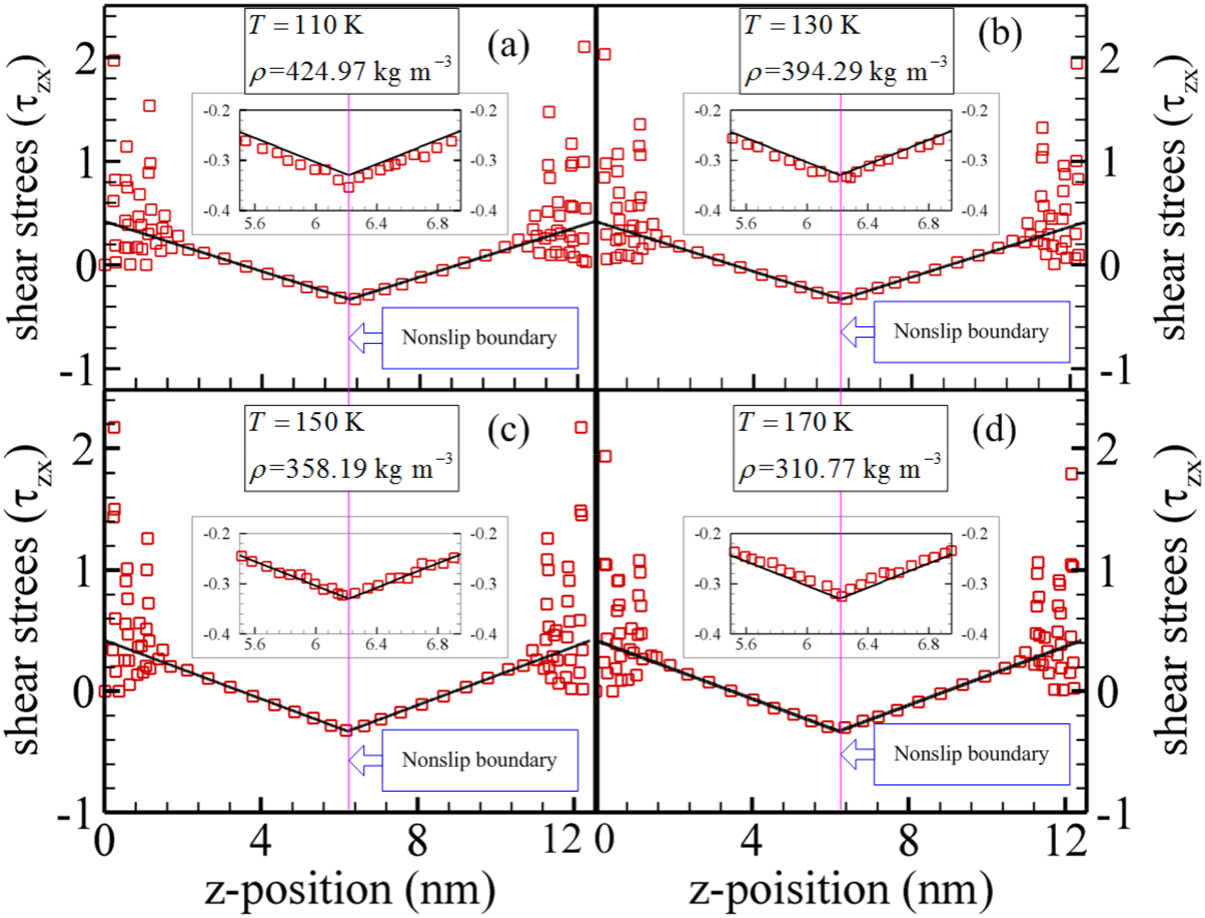
Shear stress profile from mixture boundary Poiseuille flow model via NEMSMD simulations (red squares) and theoretical value (black solid lined).

The shear stress profiles (reduced unit ${\varepsilon \mathord{\left/ {\vphantom {\varepsilon {\sigma^{3} }}} \right. \kern-\nulldelimiterspace} {\sigma^{3} }}$) are showed in Fig. 4 for selected state points based on the mixture boundary Poiseuille flow model using the NEMSMD simulations. Because of the powerful repulsion interaction force between the fluid molecule and silicon atom, the shear stress presents significant vibration for all cases in the fluid region near the nanochannel surfaces. Furthermore, with the temperature increasing, the shear stress slightly increases as the fluid molecules approach the domain of the nonslip boundary. The reason may be that the methane molecules are conducted by the same value of body driving force with opposite direction while it crosses the nonslip boundary. Besides, the thermal motion of methane molecules enhances with the system temperature increasing. However, we find that the shear stress is very close to the theoretical values inside the fluid domain between nonslip boundary and rough nanochannel wall. The similar result can be referred to the research of Backer et al.^44^ for LJ fluid.

**Figure 5:**
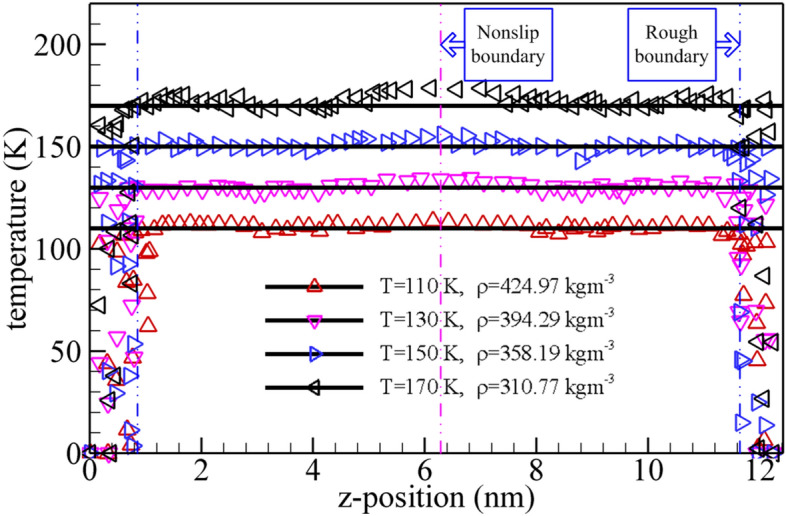
Total average temperature profiles from mixture boundary Poiseuille flow model via NEMSMD simulations (red squares) and theoretical value (black solid lined).

The temperature profiles of methane nanofluidic are calculated by the mixture boundary Poiseuille flow model using the NEMSMD simulations and plotted in Fig. 5 for different state points by Eq. (12). From the Fig. 5, the local temperature of the confined methane nanofluidic are not uniform everywhere, i.e., it shows that the fluid temperature near the nonslip boundary is higher than that of whole fluid regions. Moreover, the local temperature increases with the decreasing of the density (or increasing of the system temperature) when the fluid molecules arrive at the nonslip boundary. This reason may be that the fluid molecules are subject to the same body driving force with opposite direction while it crosses the nonslip boundary, which produces the latent heat of methane fluid molecule. Furthermore, the temperature profiles present much variation near the rough nanochannel surfaces, which are very close to the results of the DPD simulation by Kasiteropoulou et al.^30^ for the Poiseuille flow confined inside grooved nanochannel. We ascribe this reason to the intricate interaction force (between wall atom and fluid molecule) which remarkably impacts the distributions of atom number density (Fig. 3). In addition, the values of temperature also illustrated a slight oscillation around the temperature of system in the center regions between nonslip boundary and rough nanochannel surfaces. The reason may be ascribed to the matter of fact that the temperature of the simulation system is regulated by calculating the kinetic energy in y and z coordinate directions. Besides, the statistical errors are not be ignored completely. These numerical results are generally similar to those of confined fluids by the NEMD simulations^53^. All numerical simulation results for temperature profiles of methane confined fluid also demonstrate that the mixture boundary Poiseuille flow model is reasonable and credible to study the confined methane nanofluidic using NEMSMD simulations.

**Figure 6:**
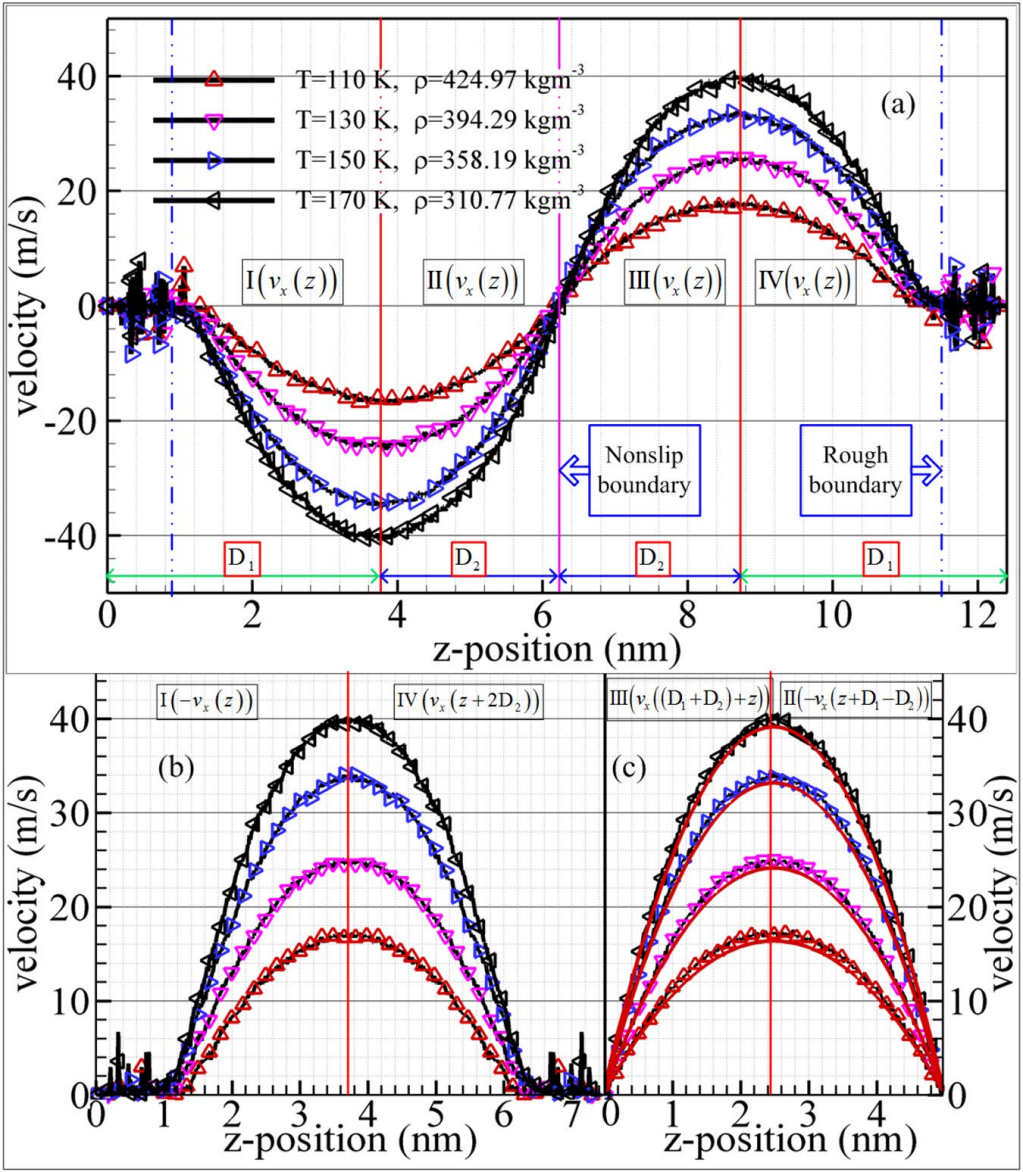
The velocity profiles from mixture boundary Poiseuille flow model by NEMSMD simulations. The black solid curves are guide. And red curve lines denote the analytical solution.

**Figure 7:**
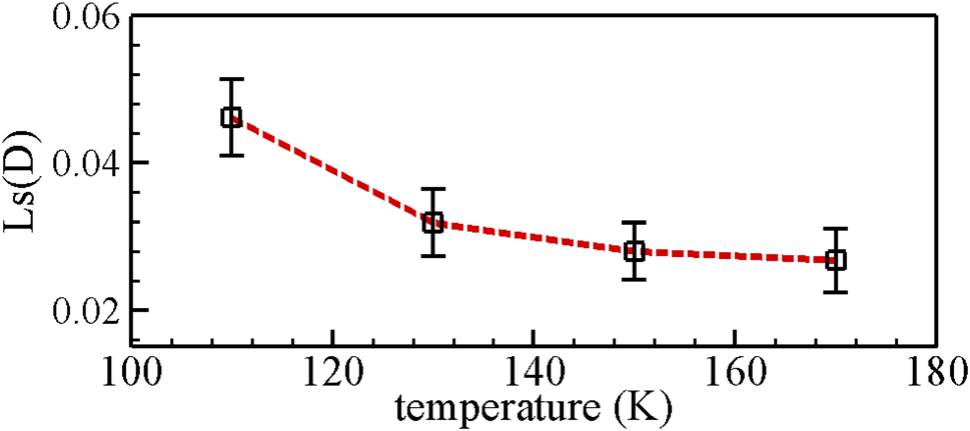
Slip lengths $L{\text{S}}\;\left( {\text{D}} \right)$ of methane nanofluidic near rough boundary and calculation errors by the presented model. The red dashed curves are guide.

In order to illustrate the influence of the complex boundary on the hydrodynamic properties for the confined methane nanofluidic, the velocity profiles of the nonslip boundary are compared with that of rough boundary based on the local symmetrical and coordinate transformation methods. Figure 6a plots the velocity profiles of methane confined nanofluidic at the selected state points. As can be seen from the Fig. 6a, the irregular fluctuation of velocity values can be clearly observed near the rough nanochannel surfaces, this reason can be ascribed to the fact that the fluid molecules are subjected to the complex interaction force between wall atom and methane molecule inside the cavities of wall. Moreover, the velocity profiles of methane nanofluidic increase with the temperature increasing (or density decreasing), the velocity profiles demonstrate the central symmetry about the point of intersection for the *z*-position line and nonslip boundary. However, the velocity profiles of ${\text{I}}\left( {v_{{\text{x}}} \left( z \right)} \right)$(${\text{III}}\left( {v_{{\text{x}}} \left( z \right)} \right)$) and ${\text{II}}\left( {v_{{\text{x}}} \left( z \right)} \right)$(${\text{IV}}\left( {v_{{\text{x}}} \left( z \right)} \right)$) are asymmetric. We ascribe it to the fluid molecule obtaining diverse interaction forces in the fluid domain nearby nonslip boundary or nature rough nanochannel surfaces. These numerical results are similar to those of the simple argon confined nanofluidic^16,18^ or the methane confined nanofluidic inside asymmetrical nanochannel surfaces with the rough upper wall and the smooth underneath wall^33,37^. Furthermore, the fluid velocity profiles of fluid regions near the rough nanochannel are showed in Fig. 6b by the local symmetrical and coordinate transformation techniques. The numerical results qualitatively agree well with our previous research results of the rough nanochannel surfaces^33^. Figure 6c plots the velocity profiles with the nonslip boundary under the selected state points. These numerical results approach the theoretical value with red lines for methane nanofluidic, and the viscosity values can be referred to the experiment^54^. Although the numerical velocities of the proposed model have a slight difference with the analytical solution by the governing equation for Poiseuille flow at the nonslip boundary: $v_{{\text{x}}} \left( z \right) = \frac{{\rho f_{{{\text{ex}}}} h}}{2\eta }\left( {\frac{1}{4} - \left( {z - \frac{1}{2}} \right)^{2} } \right)$^45^. We attribute this slight difference to methane fluid molecules migrating in z-direction and intermolecular interaction. The slip lengths of methane nanofluidic near the rough boundary are showed in Fig. 7 using equation: $L{\text{s = }}{{Vs} \mathord{\left/ {\vphantom {{Vs} {\dot{\gamma }}}} \right. \kern-\nulldelimiterspace} {\dot{\gamma }}}$ ($Vs$ and $\dot{\gamma } = {{dVs} \mathord{\left/ {\vphantom {{dVs} {dz}}} \right. \kern-\nulldelimiterspace} {dz}}$ are slip velocity and shear rate adjacent to the rough boundary, respectively.). The numerical results indicate that the slip length decreases when the temperature increases, and the gradient of slip length slightly decreases with the increasing of the temperature. The reason can be attributed to the fluid molecule suffered different interaction forces near the rough boundary at different state points, which leads to the local viscous force decreasing with the increasing of the temperature. Besides, we speculated that the discrepancy of slip length was caused by the difference in fluid viscosity at different temperatures. All above simulation results indicate that the proposed mixture boundary Poiseuille flow model is an effective and credible model for investigating the hydrodynamic properties of confined methane nanofluidic, and the validity of NEMSMD framework are also showed. To further illustrate the effect of complex boundary on hydrodynamic properties for confined methane nanofluidic, the values of shear viscosity (methane fluid) are calculated by fitting local velocity information derived from the NEMSMD simulations for different state points in the next Section.

### Shear viscosity

Table 3 shows the shear viscosity for the whole fluid conducted by nonslip and rough boundary conditions based on the proposed model with different state points (temperature and density). As it can be seen that the variation trend of shear viscosity is well displayed by the confined methane nanofluidic with the nonslip and rough boundary, the numerical results of the nonslip boundary are in better agreement with the experimental values than those of the fluid domain confined by rough boundary, which verifies the validity of the proposed model and the fitted local velocity technique in calculating shear viscosity for methane fluid. Their difference is attributed to different boundary conditions and the methane molecule model (to our knowledge, no molecule model can accurately predict all properties of methane at all state points^7,44,54^). Moreover, the slip length of methane nanofluidic near the rough boundary may be dependent on the viscosity of fluid, i.e., the slip length decreases with the increasing of the viscosity (seen from Fig. 7 and Table 3). Last but not least, it is worthy of noting that the shear viscosity of the nonslip and rough boundary conditions are in agreement with the experiment values when the temperature is bigger than $170\;{\text{K}}$(or the density is less than $310.77\;{{{\text{kg}}} \mathord{\left/ {\vphantom {{{\text{kg}}} {{\text{m}}^{3} }}} \right. \kern-\nulldelimiterspace} {{\text{m}}^{3} }}$). This fact is ascribed to the reason that the temperature (or the density) significantly influences the prediction results of shear viscosity for methane fluid. The numerical result also demonstrates the validity of fitted local velocity method in calculating the shear viscosity of confined nanofluidic.

**Table 3:** The values and relative error of viscosity for methane nanofluidic by the presented model.

State points		Nonslip boundary		Rough boundary		Experiment^54^
$T\left( K \right)$	$$\begin{gathered} \;\;\;\;\rho \hfill \\ \left( {{{kg} \mathord{\left/ {\vphantom {{kg} {m^{3} }}} \right. \kern-\nulldelimiterspace} {m^{3} }}} \right) \hfill \\ \end{gathered}$$	$$\begin{gathered} \;\;\;\;\;\eta_{{{\text{cal}}}} \hfill \\ \left( {{{\upmu {\text{g/cm}}}}\;{\text{s}}} \right) \hfill \\ \end{gathered}$$	$\frac{{\eta_{{{\text{cal}}}} - \eta_{\exp } }}{{\eta_{\exp } }}$	$$\begin{gathered} \;\;\;\;\;\eta_{{{\text{cal}}}} \hfill \\ \left( {{{\upmu {\text{g/cm}}}}\;{\text{s}}} \right) \hfill \\ \end{gathered}$$	$\frac{{\eta_{{{\text{cal}}}} - \eta_{\exp } }}{{\eta_{\exp } }}$	$$\begin{gathered} \;\;\;\;\;\eta_{\exp } \hfill \\ \left( {{{\upmu {\text{g/cm}}}}\;{\text{s}}} \right) \hfill \\ \end{gathered}$$
100	439.02	${1433} \pm 24$	$- 0.083$	$1336 \pm 14$	$- 0.145$	1563
110	424.97	${1}0{87} \pm 13$	$- 0.111$	$1078 \pm 29$	$- 0.119$	1223
120	410.13	$847 \pm 12$	$- 0.139$	$795 \pm 29$	$- 0.192$	984
130	394.29	$716 \pm 16$	$- 0.113$	$664 \pm 30$	$- 0.177$	807
140	377.15	$652 \pm 17$	$- 0.025$	$613 \pm 27$	$- 0.084$	669
150	358.19	$496 \pm 18$	$- 0.111$	$477 \pm 21$	$- 0.145$	558
160	336.59	$454 \pm 18$	$- 0.022$	$430 \pm 18$	$- 0.073$	464
170	310.77	$396 \pm 15$	$0.042$	$384 \pm 22$	$0.011$	380
180	276.58	$299 \pm 13$	$- 0.003$	$301 \pm 17$	$0.003$	300
190	200.20	$179 \pm 12$	$- 0.043$	$199 \pm 15$	$0.064$	187

**Figure 8:**
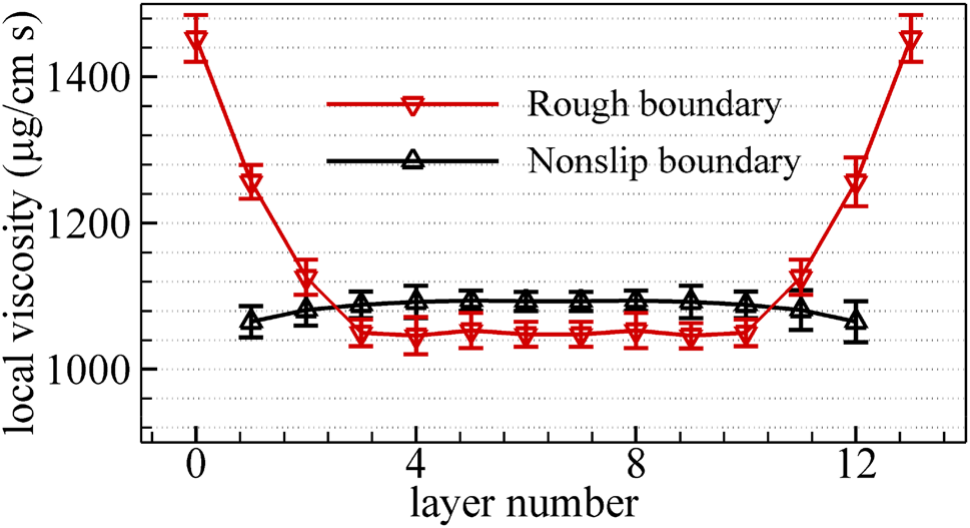
Comparison of the values of local shear viscosity of the nonslip boundary with that of the rough boundary. The viscosity values are calculated by fitting the local velocity information as obtained from the mixture boundary Poiseuille flow model ($T = 110\;{\text{K,}}\;\rho = {424}{\text{.97}}\;{\text{kg}}\,{\text{m}}^{ - 3}$). The calculation errors bar for local viscosity are also shown.

Furthermore, to better evaluate the influence of the boundary condition on the internal frictional force for confined methane nanofluidic, the local shear viscosity in different fluid layers is further depicted in Fig. 8. By comparing the local shear viscosity of the nonslip boundary with that of the rough boundary condition, it is observed that the present results regarding the local shear viscosity approach the experiment data for saturated liquid methane in the middle of fluid regions. The reason may be that the boundary conditions (nonslip boundary or rough boundary) obviously impact on the mobility of fluid molecule when the fluid molecule crosses the fluid region near the boundary, as are also shown by the atom number density distribution profiles in Fig. 3, stress profiles in Fig. 4 and velocity profiles in Fig. 6. In addition, the values of shear viscosity rapidly aggrandizes in the fluid nearby the rough nanochannel surfaces, which qualitatively agree with the simulation results for fluid confined in rough nanochannel walls^10,17^. And it differs significantly either in the nonslip boundary, as a matter of fact, the shear viscosity values slightly reduce in the fluid layer near the nonslip boundary, which is ascribed to the diminution of fluid layer molecule density (seen form Fig. 3). The numerical result also indicate that the shear thinning occurs for the near nonslip boundary. It indicate the shear rate increases at positions of the fluid domain approaching nonslip boundary. The reason can be ascribed to the fact that the methane fluid molecule are conducted by the same value of body driving force with opposite direction while it crosses the nonslip boundary. Last but not least, maximum viscosity values are demonstrated in the fluid domain adjacent to the rough nanochannel surfaces, while the viscosity values of methane nanofluidic interior are almost similar for the rough wall investigated here. In the fluid domain of the nonslip boundary, minimum values of viscosity are observed near the nonslip boundary domain, while viscosity values in the interior region are almost similar. These numerical results indicate that the mixture boundary Poiseuille flow model could well be used to investigate the influence of the nonslip and natural physical (silicon atomic plates) boundary conditions on hydrodynamic properties of methane nanofluidic in one NEMSMD simulation.

### Molecular mechanism

**Figure 9:**
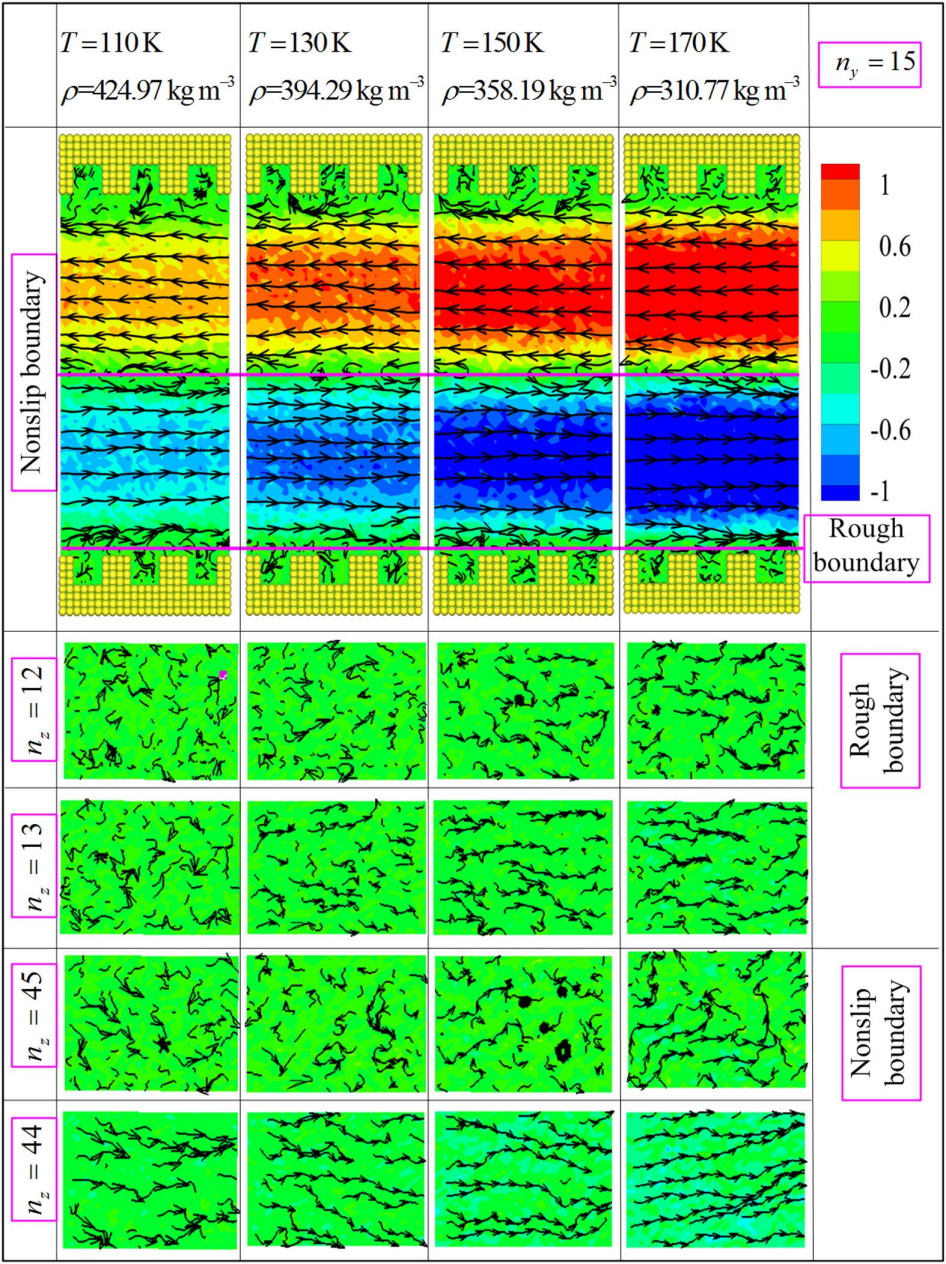
The streamlines and mainstream velocity $v_{x} \left( z \right)$ (the unit of velocity is $\left( {{\varepsilon \mathord{\left/ {\vphantom {\varepsilon m}} \right. \kern-\nulldelimiterspace} m}} \right)^{1/2}$) contour of methane fluid for selected state point using the mixture boundary Poiseuille flow model.

To obtain deeper understanding of the hydrodynamic properties dependence for the methane transport in nanochannel, we further investigate molecular mechanism of methane fluid flowing with nonslip and natural physical (silicon atomic plates) boundary conditions. However, there is no existing numerical and experimental data of the nanochannel confined flow for comparisons nonslip and rough boundary conditions in one simulation (the effect of the nonslip and rough boundary on the nanofluidic behaviors) so far. Figure 9 displays the streamline and dimensionless mainstream velocity (the unit of velocity is $\left( {{\varepsilon \mathord{\left/ {\vphantom {\varepsilon m}} \right. \kern-\nulldelimiterspace} m}} \right)^{1/2}$) contour of methane molecule using the proposed mixture boundary at the selected state points. It is observed that the streamlines are significantly distorted as the molecule is closed to the nonslip boundary and rough nanochannel surfaces. Actually, this flow behavior is that the methane molecule suffers from complex interaction force near the boundary regions. The fluid molecule is mainly subjected to wall atom interaction force in the fluid domain near the rough nanochannel surfaces. In the fluid domain of the nonslip boundary, however, the molecular movement of methane are significantly manipulated by the opposite driving force and intermolecular forces. As one can see from the bottom of Fig. 9 ($n_{z} = 13,\;n_{z} = 44$), the disorder level of streamlines increases with the decreasing of temperature near the fluid domain for the rough and nonslip boundary. And the streamlines disorder level of near nonslip is more obvious than that of rough boundary. It indicates that the movement of fluid molecules near the boundary is not only manipulated by the velocity in x direction, but also the velocities in y and z direction should be considered. The impact of y and z directions' velocities on the movement fluid of molecules gradually increases with the decreasing of the distance between wall atom and fluid molecule. Besides, The impact of y and z directions' velocities on the movement fluid molecules nearby the rough boundary is larger than the nonslip boundary. These results demonstrate that the rough boundary has more significant influence on the molecular movement for confined nanofluidic than nonslip boundary. The above results of streamlines present that the velocity field suffer from the influence of boundary significantly, because of the complex interaction force near the boundary. Furthermore, as the temperature rises (or density decreases), the absolute value of mainstream velocity increases in the fluid region between nonslip boundary and rough nanochannel surfaces. It is attributed to two factors: the thermal motion of methane molecule increases when the temperature rises (or density decreases), and the viscosity of fluid decreases with the increasing of the temperature (decreasing of the density).

**Figure 10:**
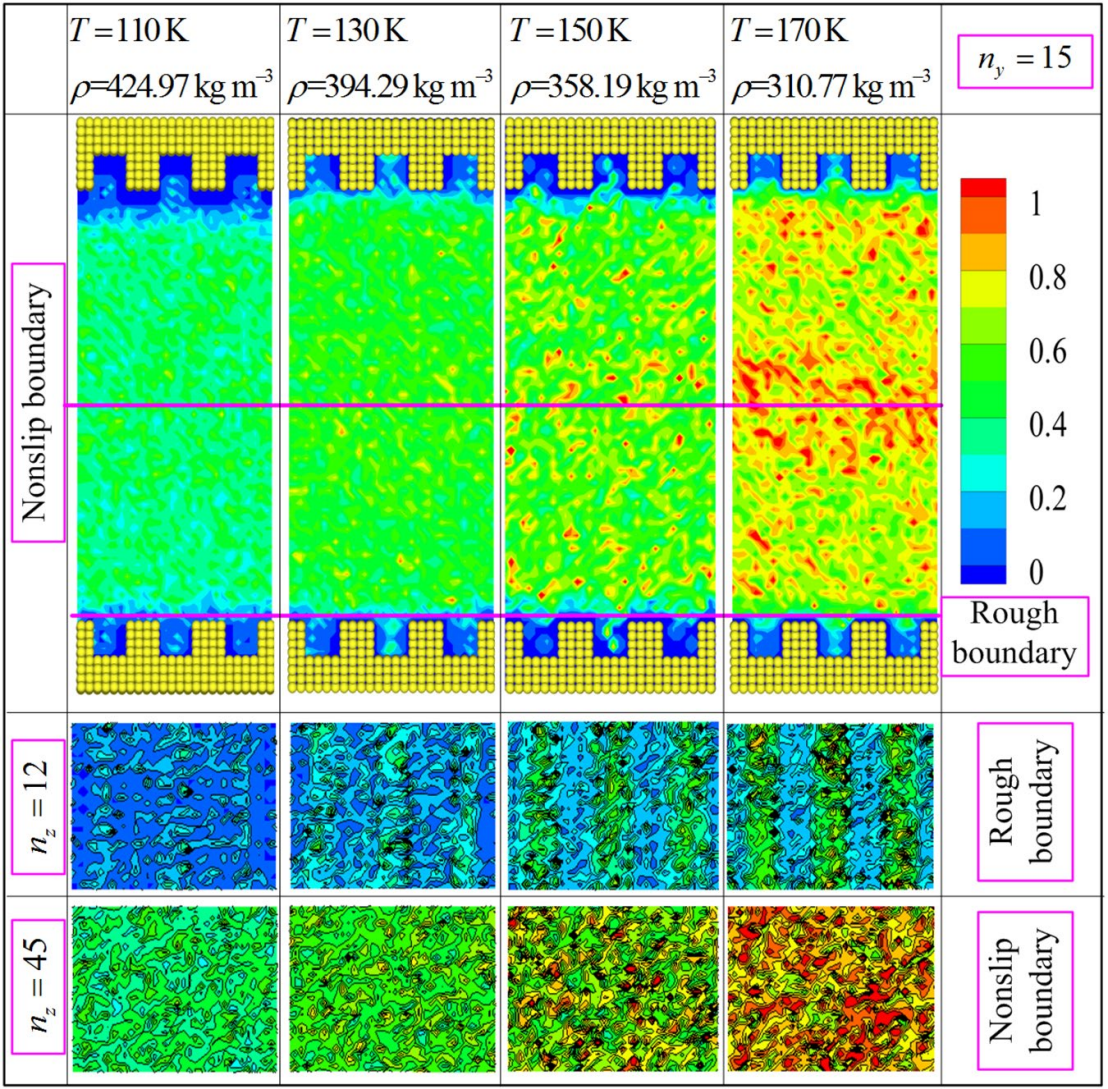
Rotational energy (the unit of energy is $\varepsilon$) contour plots for different state points using the mixture boundary Poiseuille flow model.

**Figure 11:**
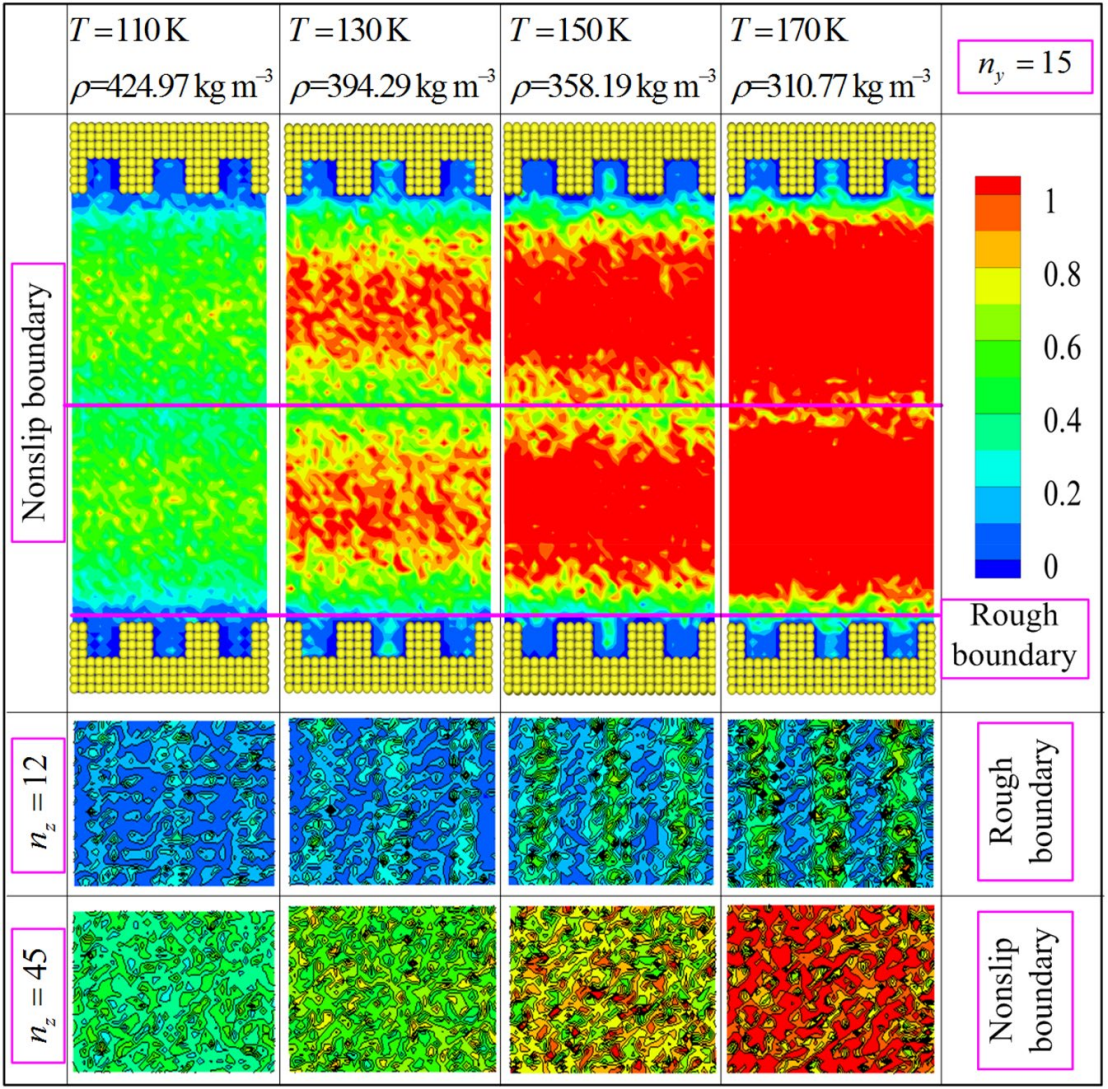
Translational energy (the unit of energy is $\varepsilon$) contour plots for different state points using the mixture boundary Poiseuille flow model.

In addition, the above numerical results are mainly attributed to the rotational movement and translational mobility of methane molecules confined by the nonslip and rough boundary conditions. To verify and examine this point, the contour distributions of rotational and translational energies calculated via ensemble average are plotted in Figs. 10 and 11 based on the proposed model. As seen in Figs. 10 and 11, the smallest values of rotational and translational energies ($n_{z} = 12$) are observed near the rough nanochannel surfaces. The result is attributed to the reason that the fluid molecule obtained stronger constraining force from wall atom when it flow the domain near the nanochannel surfaces. And this constraining force slows down the translational motion and rotary movement of methane molecule. Actually, in the fluid region of near nanochannel surfaces ($\le r_{{{\text{cut}}}}$), the interaction force of fluid molecules is conducted by MOPLS model ($U_{{{\text{MOPLS}}}} \left( {r_{ij} } \right)$), and that between wall atoms and fluid molecules is determined using the wall–fluid interaction potential ($U_{{{\text{MS}}}} \left( {r_{ij} } \right)$). However, Fig. 10 shows that the maximum values of rotational energy obviously observed near the nonslip boundary ($n_{z} = 45$) at state point ($T = 170\;{\text{K}}$ and $\rho = 310.77\;{\text{kg}}\,{\text{m}}^{ - 3}$). This result is attributed to the moving in opposite directions of fluid molecules in the two sides of the nonslip boundary, leading to the thermal motion of molecules increasing. Moreover, Figs. 10 and 11 ($n_{z} = 12$) show that the rotational and translational energies present the periodic variation along the x direction. The periodicity of the rotational and translational energies strictly depend on the geometrical configuration of rough nanochannel surfaces, and the peak value's position of the rotational and translational energies approach to the middle of the cavity of nanochannel walls. Furthermore, in the whole fluid region, Figs. 10 and 11 present the rotational and translational kinetic energy which increase with the increases of the temperature (or decreasing of density). We attribute to the rotational movement of fluid molecules stronger dependent on the state point, i.e., the higher the temperature is, the severer the rotational movement is. Furthermore, Fig. 11 presents that the translational kinetic energy shows the symmetry with nonslip boundary from the whole simulation domain, indicating the absolute value of velocity is almost symmetry with nonslip boundary. The contour of translational kinetic energy illustrates the asymmetric in the fluid region between nonslip boundary and rough boundary, which demonstrate that the different boundary conditions obviously influence on the translational kinetic energy of fluid molecule. Besides, the local rotational energy and translational kinetic energy obviously differ in the region near the nonslip and rough boundary conditions. The mainly reason ascribed to the fluid molecule suffered different interaction forces when it moves to the fluid regions near the nonslip and nature rough boundary. It is worthy of noting that the translational kinetic energy arrives at minimal value in fluid region near the nonslip boundary (the picture is also showed in Fig. 10). This result also indicates that the fluid molecules have stronger thermal motion near the nonslip boundary region than other fluid region. In nanoscale, the complex interaction force is the primary cause impacting on the hydrodynamic properties of confined fluid molecule. These numerical results demonstrate that the hydrodynamic characteristics of confined fluid strictly relay on the movement of fluid molecule, including the rotational motion and translational motion. In fact, the hydrodynamic characteristics of the confined fluid are strictly related to the state point of fluid and boundary condition. Therefore, the proposed model is credible to study the hydrodynamic properties of methane confined nanofluidic by the NEMSMD simulation in the extreme boundary condition (nonslip and rough boundary conditions).

## Conclusions

The mixture boundary Poiseuille flow model is proposed to explore the influence of the rough and the nonslip boundary on hydrodynamic properties for methane confined nanofluidic in one NEMSMD simulation. All numerical results demonstrate that our proposed model is effective and credible scheme to study the extremely boundary confined fluid. The most major findings are obtained from this study also including:

1. The distance of between two rough nanochannel walls is larger than 12 molecules diameter (about $4.968\,{\text{nm}}$) if you want to calculate the viscosity by the fitted velocity profile method using the proposed modeling.
2. The number density distributions of C and H atoms obviously differ with nonslip and rough boundary conditions, including the difference of distribution for stress, temperature and velocity which indicate the methane fluid molecules suffered from different interaction force when the methane molecule is close to the conducted domain of the nonslip or rough boundary conditions.
3. The influence of the nonslip boundary on the shear viscosity is less than that of rough boundary.
4. Near the nonslip boundary, the local viscosity value (atom (C and H) density) is less than that of other fluid layer, indicating the shear thinning of fluid.
5. The slip length of methane nanofluidic near the rough boundary decreases with the increasing of the temperature.
6. In the fluid domain near the boundary, the streamlines are distorted. Moreover, the rough boundary has more significant influence on the molecular movement for confined nanofluidic than nonslip boundary.

This study will provide a novelty understanding for the hydrodynamic behavior of methane nanofluidic with the nonslip and rough boundary conditions, and contribute to further theoretical research for methane nanofluidic evaluation and exploitation. The extension of this study for NEMSMD simulation according our previous works^9–11,22^ affords researchers and engineers a good choice for simulations. Next work, the proposed model will be used to study the shale gas, water and methane mixture system, haze aerosol, the microelectronic design of nano-devices, and so on.

## Data Availability

All data generated or analyzed during this study are included in this published article.
